# A CD44v^+^ subpopulation of breast cancer stem-like cells with enhanced lung metastasis capacity

**DOI:** 10.1038/cddis.2017.72

**Published:** 2017-03-16

**Authors:** Jing Hu, Gang Li, Peiyuan Zhang, Xueqian Zhuang, Guohong Hu

**Affiliations:** 1 The Key Laboratory of Stem Cell Biology, Institute of Health Sciences, Shanghai Institutes for Biological Sciences, Chinese Academy of Sciences& Shanghai Jiao Tong University School of Medicine, University of Chinese Academy of Sciences, Shanghai, China

## Abstract

Cancer stem-like cells (CSCs) are a subpopulation of cancer cells responsible for tumor growth, and recent evidence suggests that CSCs also contribute to cancer metastasis. However, the heterogeneity of CSCs in metastasis capacities is still unclear in breast cancer. Here we show that among the CD24^−^/CD44^+^ breast CSCs, a subset expressing the variant isoform of CD44 (CD44v) displays significantly higher capacity of lung metastasis than that expressing the standard CD44 isoform CD44s. Increasing or reducing the CD44v/CD44s ratio of breast cancer cells by regulating the expression of epithelial splicing regulatory protein 1 (ESRP1) leads to promotion or suppression of lung metastasis without influencing cancer cell stemness. Directly suppressing CD44v expression significantly alleviates the metastasis burden in lungs. Mechanically, CD44v, but not CD44s, responds to osteopontin (OPN) in the lung environment to enhance cancer cell invasiveness and promote lung metastasis. In clinical samples expression of ESRP1 and CD44v, rather than CD44s or total CD44, positively correlates with distant metastasis. Overall, our data identify a subset of metastatic breast CSCs characterized by CD44v expression, and suggest that CD44v and ESRP1 might be better prognosis markers and therapeutic targets for breast cancer metastasis.

Heterogeneity is one of the features of malignancies rendering cancer refractory to treatment. The CSC model was proposed to explain cancer cell heterogeneity decades ago, but became prevailing only recently.^1, 2^ CSCs, sometimes also named as cancer stem cells or tumor-initiating cells, are a subset of tumor cells defined by their capacity to self-renew and differentiate into cells without tumorigenicity ability.^3^ Being first identified in acute myeloid leukemia,^4^ CSCs were also found in many solid tumors, including breast cancer,^5, 6, 7^ colon cancer,^8, 9, 10, 11^ prostate cancer,^12^ ovarian cancer,^13, 14, 15, 16^ pancreatic cancer,^17^ glioblastoma,^18^ brain tumors,^19, 20^ osteosarcoma,^21^ chondrosarcoma,^22^ gastric cancer,^23^ melanoma^24^ and lung cancer.^25^ Accumulating evidence demonstrates that CSCs not only are responsible for tumor initiation and recurrence after chemotherapy, but also contribute to distant metastasis of cancer. In breast cancer, CSCs display enhanced capacities of *in vitro* invasiveness and *in vivo* metastasis as compared to non-CSCs. In addition, higher CSC contents in breast tumors link to poor prognosis and distant metastasis.^26, 27, 28, 29^

Although an overall metastatic property has been linked to cancer stemness, CSC itself might not be homogeneous in the capacity of metastasis. Indeed, a few previous studies have demonstrated that distinct subsets of CSCs determined tumor growth and metastasis in pancreatic cancer^30^ and colorectal cancer.^31, 32^ The studies showed that only a subset of CSCs, namely metastatic CSCs, give rise to metastasis. The identification of metastatic CSCs is of clinical importance as targeting this subpopulation may be more efficient to eliminate metastasis. However, metastatic CSCs have not been reported in breast cancer, and the exact role of CSCs in breast cancer metastasis is still unclear.

CD44 is a transmembrane glycoprotein involved in many cellular processes, including cell division, survival, migration and adhesion.^33^ Since the identification of CSCs in solid tumors,^5^ CD44 has been widely used as a CSC marker in breast cancer^5^ and other malignancies.^8, 17, 23, 34, 35, 36^ The human *CD44* gene is located on chromosome 11p13 and encodes a polymorphic group of proteins (85–250 kDa in size) via alternative splicing mediated by epithelial splicing regulatory proteins (ESRPs).^37, 38^ The standard CD44 isoform CD44s includes only constitutive exons, while the variant CD44v isoforms contain one or more variable exons. Accumulating evidence implies that CD44s and CD44v might play different roles in physiology and pathology, and cancer cells often express large CD44v.^37^ However, the function of CD44v in cancer progression and metastasis is still ambiguous. In this study, we demonstrated the heterogeneity of CSCs expressing different CD44 isoforms in breast cancer, and identified a CSC subpopulation with enhanced lung metastasis capacity.

## Results

### A subpopulation of breast CSCs with enhanced lung metastatic capacity

**Figure 1:**
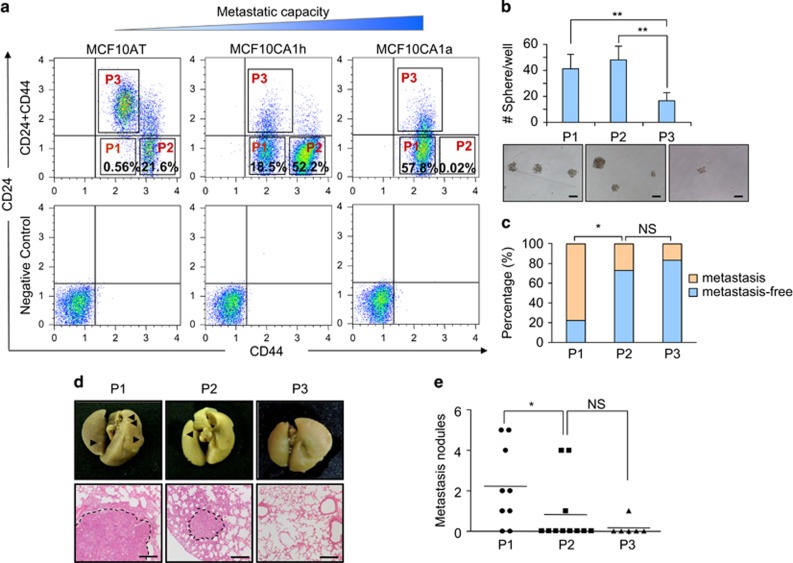
Breast CSCs are heterogeneous with variable metastatic abilities. (**a**) CSC subpopulations defined by CD24 and CD44 expression in MCF10 cell lines, *n*=3. (**b**) Quantitation and representative images of tumorspheres in MCF10CA1h subpopulations, results are expressed as mean±SD, *n*=3. (**c–e**) Lung metastasis analysis by orthotopic injection of MCF10CA1h subpopulations (*n*≥6 in each group). The data shown were mouse percentages with or without lung metastasis (**c**), representative images of lung metastases (**d**), and quantitation of metastasis nodules (**e**). Arrowheads denote the metastasis nodules on the lung surface; dotted line areas denote metastasis areas. Scale bars, 100 *μ*m (**b**), 200 *μ*m (**d**). *P<0.05; **P<0.01; NS, not significant

To study the relationship of CSCs and metastasis in breast cancer, we analyzed CSC contents of the isogenic MCF10 cancer cell lines by cell flow cytometry (FACS) with the prevailing markers CD24 and CD44. These cell lines, including MCF10AT, MCF10CA1h and MCF10CA1a, displayed gradually increasing malignancy and produced in xenografts benign hyperplasia progressing to carcinomas, largely well-differentiated carcinomas but mixed with undifferentiated areas, and poorly differentiated carcinomas with lung metastases, respectively.^39, 40^ It was observed that the CD24^-^/CD44^+^ population in these cell lines divided into two subpopulations with apparently different CD44 staining intensities, CD24^-^/CD44^med^ (referred as P1 thereafter) and CD24^-^/CD44^hi^ (P2), although both subpopulations were CD44 positive. Interestingly, only the P1 content, but not that of P2 or the overall CD24^-^/CD44^+^ population, increased along with the metastatic capacity of the cell lines (Figure 1a). So, we hypothesized that, P1, but not P2, was enriched with CSCs with metastatic ability. In order to test the hypothesis, we first analyzed the stemness of these two subpopulations. The subpopulations of CD24^-^/CD44^med^ (P1), CD24^-^/CD44^hi^ (P2) and CD24^+^/CD44^med^ (P3) were isolated from MCF10CA1h cells, and analyzed via *in vitro* tumorsphere assays and *in vivo* limiting dilution tumorigenesis assays. Compared with the non-CSC P3 cells, P1 and P2 formed significantly more tumor spheres (Figure 1b), and displayed higher tumor-initiating abilities in NOD/SCID mice. Orthotopic injection of P1 and P2 for as few as 200 cells produced primary tumors in mice, whereas in most mice, 10 000 P3 cells were required for tumor formation (Table 1 and Supplementary Figure 1). Thus, both P1 and P2 were enriched of CSCs, although P2 cells displayed slightly higher tumorigenicity than P1 at lower concentrations (Supplementary Figure 1).

We then assessed lung metastasis of the mice with primary tumors of P1, P2 or P3, and found that P1 tumors were much more metastatic than P2 and P3 tumors. Eighty percent of the mice with P1 primary tumors developed lung metastases, while only 27.3% and 16.7% of P2 or P3 tumors led to lung metastases (Figure 1c). In addition, more tumor nodules were observed on the lung surface in the P1 group than in the other groups (Figures 1d and e). Notably, the difference of P2 and P3 tumors in lung metastasis was not significant (Figures 1c and e). These data suggested that only the P1 population was enriched with metastatic CSCs.

### The metastatic CSCs express CD44v

**Figure 2:**
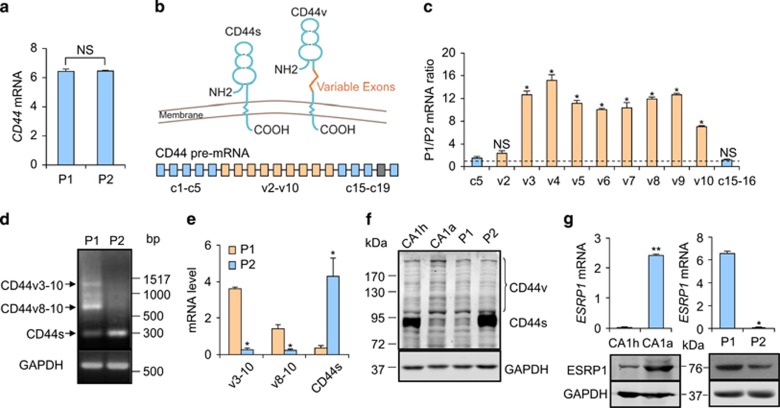
Lung-metastatic CSCs predominantly express CD44v isoforms. (**a**) Expression of total CD44 in MCF10CA1h CSC subpopulations, results are expressed as mean±S.D., *n*=3. (**b**) Schematic of different CD44 isoforms. (**c**) Expression ratios of each CD44 exons in MCF10CA1h CSC subpopulations, results are expressed as mean±S.D., *n*= 3. Dotted line define mRNA ratio of 1. (**d**,**e**) Expression of different CD44 isoforms in MCF10CA1h CSC subpopulations. Shown were electrophoresis image of RT-PCR, *n*= 4. (**d**) and qPCR data, results are expressed as mean±S.D., *n*=3. (**e**). (**f**) Protein expression of different CD44 isoforms in MCF10CA1h, MCF10CA1a and MCF10CA1h CSC subpopulations. (**g**) ESRP1 expression in MCF10CA1h, MCF10CA1a and MCF10CA1h CSC subpopulations, results are expressed as mean±S.D., *n*=3. *P<0.05; **P<0.01; NS, not significant

Next we sought to analyze the marker difference of the two CSC subpopulations, P1 and P2. Although FACS analysis suggested that P2 had a stronger CD44 signal, qPCR analysis with primers of CD44 constitutive exon regions showed that P1 and P2 had comparable CD44 expression levels (Figure 2a). Hence, we speculated that the different CD44 signal strengths of P1 and P2 in FACS assays could be caused by the expression of distinct CD44 isoforms. Human CD44 pre-mRNA consists of 19 coding exons, including 9 variable exons (v2-v10, and v1 is not expressed in human CD44) and 10 constitutive exons (c1–c5 and c15–c19).^38^ Alternative splicing of CD44 pre-mRNA by ESRPs generates the CD44s isoform containing only the constitutive exons and a list of CD44v isoforms containing the constitute exons and different numbers of variable exons.^37^ CD44v isoforms have an enlarged stem structure compared to CD44s (Figure 2b). To discriminate the isoforms P1 and P2 preferentially expressed, we performed CD44 exon-specific qPCR in the two subpopulations. The expression levels of constitutive exons in P1 and P2 were comparable, while P1 expression of the variable exons was significantly higher (Figure 2c), suggesting upregulation of CD44v in P1. Consistently, RT-PCR followed by gel electrophoresis also showed that P1 preferentially expressed the CD44v isoforms containing variable exons 3–10 (CD44v3–10) and variable exons 8-10 (CD44v8–10), and P2 predominantly expressed CD44s (Figure 2d). This result was further validated at the mRNA and protein levels by CD44 isoform-specific qPCR and western blots (Figures 2e and f). In addition, MCF10CA1h and MCF10CA1a preferred to express CD44s and CD44v, respectively, a phenotype concordant to their different contents of P1 and P2 subpopulations (Figure 2f). Given that ESRP1 regulates CD44 alternative splicing,^38^ we also detected *ESRP1* expression in MCF10CA1h, MCF10CA1a, as well as in P1 and P2 subpopulations. MCF10CA1a and P1 expressed ESRP1 more abundantly than MCF10CA1h and P2, respectively (Figure 2g). In conclusion, the P1 metastatic subpopulation of CSCs is characterized by the preference to express CD44v3–10 and CD44v8–10 isoforms.

We further analyzed additional breast cancer cell lines, SCP28, MDA-MB-231 and BT20, for the expression of CD44v in CD24^-^/CD44^+^ population. Although there was no distinct subpopulation separation in these cell lines as in MCF10 cells, CD44v^-^ and CD44v^+^ cells were also observed in the CD24^-^/CD44^+^ populations of these cell lines (Supplementary Figure 2). Notably, the metastatic subline SCP28 contained more CD24^-^/CD44^+^/CD44v^+^ cells (3.54%) than its parental line MDA-MB-231 (1.11%).

### ESRP1-modulated CD44 isoform switching promotes lung metastasis without changing stemness of breast cancer cells

**Figure 3:**
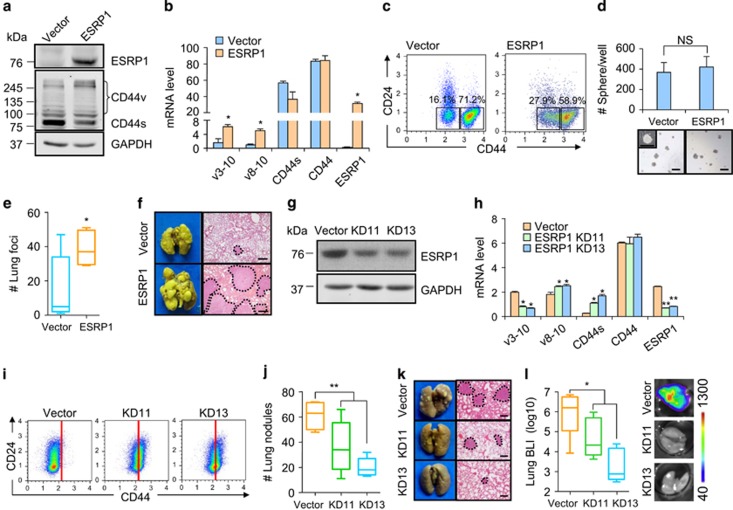
ESRP1-modulated CD44v isoform switching promotes lung metastasis without affecting cancer cell stemness. (**a**) Protein levels of ESRP1 and CD44 isoforms in MCF10CA1h after *ESRP1* overexpression. (**b**) mRNA levels of CD44 isoforms in MCF10CA1h after *ESRP1* overexpression, results are expressed as mean±SD, *n*=3. (**c**) CSC subpopulation shift in MCF10CA1h after *ESRP1* overexpression. (**d**) Tumorsphere formation in MCF10CA1h after *ESRP1* overexpression, results are expressed as mean±SD, *n*=3. (**e**,**f**) Lung metastasis after intravenous injection of MCF10CA1h with *ESRP1* overexpression (*n*=10 in each group). Quantitation of metastasis foci in lung sections (**e**) and representative images of lung metastases. (**f**) were shown. Dotted lines denote areas of lung metastases. (**g**) *ESRP1* knockdown in MCF10CA1a. (**h**) mRNA expression of *ESRP1* and *CD44* isoforms in MCF10CA1a after *ESRP1* knockdown, results are expressed as mean±SD, *n*=3. (**i**) CSC subpopulation shift in MCF10CA1a after *ESRP1* knockdown. (**j**,**k**) Lung metastasis after intravenous injection of MCF10CA1a with *ESRP1* knockdown (*n*≥6 in each group). Quantitation of lung surface nodules (**j**) and representative images of lung metastases. (**k**) were shown. (**l**) Lung metastasis after orthotopic injection of MCF10CA1a with *ESRP1* knockdown (*n*≥5 in each group). Scale bars, 300 *μ*m (**d**), 1000 *μ*m (**f**,**k**). *P<0.05; **P<0.01; NS, not significant

Then we examined whether altering CD44 isoform expression in breast cancer cells would affect lung metastasis. Given that ESRP1 regulates CD44 alternative splicing without changing the overall expression level of CD44 protein,^38^ we regulated CD44 isoform switching by modulating ESRP1 expression, in order to study the influence of CD44 isoforms on metastasis behavior of CSCs without altering the cell stemness. ESRP1 overexpression in MCF10CA1h led to the increase of CD44v expression and the decrease of CD44s expression (Figures 3a and b). FACS analysis also showed a shift of the CD24^-^/CD44^+^ cell population from P2 to P1 (Figure 3c). Tumorsphere assays demonstrated that *ESRP1* overexpression had no influence on cancer stemness of the cells (Figure 3d). However, *ESRP1*-mediated CD44 isoform switching significantly promoted lung metastasis of MCF10CA1h cells when intravenously inoculated into the mice, as revealed by elevated numbers and sizes of metastasis nodules on the lung surface, and increased weight of the lungs (Figures 3e, f and Supplementary Figures 3A and 3B). We also repeated the assays in the SCP28 breast cancer cell line and found that *ESRP1* overexpression (Supplementary Figure 3C) also led to CD44 isoform switching and elevation of lung metastasis, but not affecting the CSC feature of the cells(Supplementary Figures 3D-3G).

Next, we silenced *ESRP1* with two short hairpin RNAs (shRNAs) in MCF10CA1a and observed the CD44v-to-CD44s isoform switching, and the CSC population shift from CD44^med^ to CD44^hi^ (Figures 3g and i). Intravenous injection of MCF10CA1a into athymic mice revealed the diminished ability of the cells for lung colonization after *ESRP1* knockdown (Figures 3j, k and Supplementary Figure 3H). We also analyzed the metastasis capability of MCF10CA1a in the spontaneous metastasis model by orthotopic MCF10CA1a injection into NOD/SCID mice. Again, it was observed that both *ESRP1* shRNAs significantly repressed the lung metastasis burden of the mice (Figure 3l). Hence, ESRP1-induced CD44v isoform splicing in CSCs promotes lung metastasis without changing cancer stemness of the cells.

### OPN promotes cancer cell metastasis to lung through CD44v

**Figure 4:**
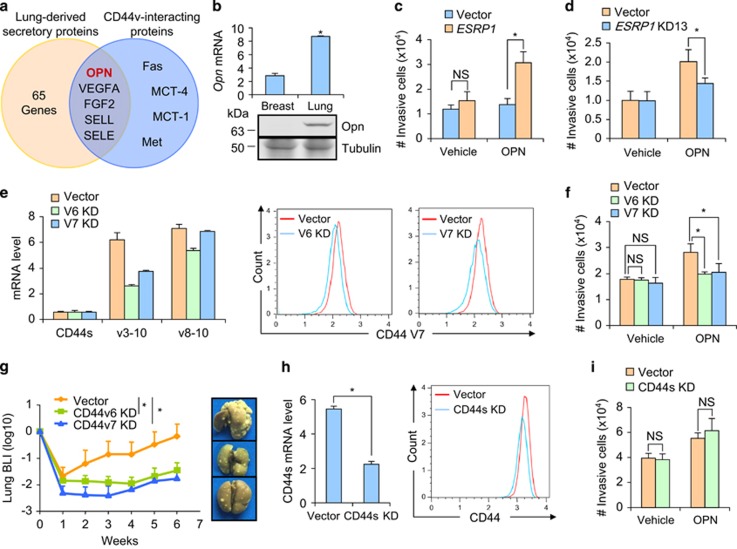
OPN promotes cancer cell invasiveness via CD44v. (**a**) Schematic of overlap analysis for proteins secreted in lung tissues and interacting with CD44v. (**b**) The expression of *Opn* in mouse breast and lung tissues, results are expressed as mean±S.D., *n*=3. (**c**) MCF10CA1h invasion after *ESRP1* overexpression with or without the treatment of recombinant OPN (5 *μ*g/ml), results are expressed as mean±SD, *n*=4. (**d**) MCF10CA1a invasion after *ESRP1* knockdown with or without the treatment of OPN (5 *μ*g/ml), results are expressed as mean±S.D., *n*=4. (**e**) CD44 isoform expression in MCF10CA1a with *CD44v6* or *CD44v7* knockdown (KD), results are expressed as mean±S.D., *n*=3. Shown on right is the FACS analysis of CD44v7 protein expression. (**f**) MCF10CA1a invasion after *CD44v6* or *CD44v7* knockdown, with or without the treatment of OPN (5 *μ*g/ml), results are expressed as mean±S.D., *n*=4. (**g**) Lung metastasis of mice with intravenous injection of MCF10CA1a with *CD44v6* or *CD44v7* knockdown, (*n*≥8 in each group). (**h**) qPCR analysis of CD44s mRNA expression, results are expressed as mean±SD, *n*=3 (left) and FACS analysis of CD44 protein expression (right) in MCF10CA1a after *CD44s* knockdown. (**i**) MCF10CA1a invasion after *CD44s* knockdown, with or without the treatment of OPN (5 *μ*g/ml), results are expressed as mean±SD, *n*=4. *P<0.05; NS, not significant

As P1 and P2 cells displayed different capabilities for lung metastasis but not tumor initiation at the primary site, it is likely that CD44v, a transmembrane protein, regulates CSC metastasis by interacting with extracellular factors in the lung microenvironment. To search for such factors, we analyzed the lists of secreted proteins of lung tissues^41^ and CD44v-interacting proteins^42, 43, 44, 45, 46, 47^ identified by previous reports. The analysis resulted in 5 proteins in the overlap of the two lists (Figure 4a). Among these 5 proteins, E-selectin (SELE) and L-selectin (SELL) were actually not enriched in lungs as compared to breast tissues (Supplementary Figure 4A-4B). Although VEGFA and FGF2 were moderately enriched in lung tissues, we did not observe the previously reported enhancement of proliferation or survival following VEGFA and FGF2 treatment in MCF10CA1h cells (data not shown). Therefore, OPN was the only candidate.

OPN is a secreted non-collagenous, sialic-acid-rich, chemokine-like protein and has been reported to involve in tumor progression and cancer cell metastasis. In addition, it is known that OPN can bind directly to CD44v on the areas of exons V3, V6 and V7.^48, 49^ We observed that lung tissues expressed OPN in a level significantly higher than in breast tissues (Figure 4b). In addition, OPN promoted cancer cell invasiveness in an ESRP1-dependent manner. Only the *ESRP1*-overexpressing MCF10CA1h cells, but not the control cells, could respond to OPN treatment and displayed significantly enhanced invasiveness (Figure 4c). Reciprocally, *ESRP1* knockdown in MCF10CA1a resulted in a subdued response to OPN for promotion of cell invasion (Figure 4d). It is also reported that OPN can suppress cancer cell apoptosis.^49^ However, we found that OPN-mediated cell survival was minor and also independent of *ESRP1* expression in MCF10CA1h (Supplementary Figure 5).

To directly elucidate the role of CD44v in OPN-mediated cancer cell invasion, we knocked down CD44v with shRNAs targeting exons V6 and V7 in MCF10CA1a, and as expected, the shRNAs suppressed the expression of CD44v3–10, but not CD44v8–10 or CD44s (Figure 4e). In accordance to the observation in cancer cells with *ESRP1* overexpression and knockdown, V6 and V7 shRNAs significantly reduced MCF10CA1a invasion in the presence of OPN (Figure 4f). In addition, CD44v knockdown in *ESRP1*-overexpressing cells diminished the effect of ESRP1 in promotion of cell invasiveness (Supplementary Figure 6A). We also tested the effect of *CD44v* knockdown in lung metastasis. When MCF10CA1a was injected into athymic mice, it was found that both shRNAs significantly suppressed the lung metastasis burden of the mice (Figure 4g). As a comparison, we specifically knocked down *CD44s* with a shRNA construct targeting the c5–c15 splicing junction area (Figure 4h) and found that CD44s suppression had no effect on cell invasiveness in the presence or absence of OPN (Figure 4i). Taken together, these data indicated that CD44v, but not CD44s, responded to OPN in lungs to promote tumor invasion and lung colonization. We further found that it was the V3–V7 exon region of CD44v to interact with OPN for cancer cell invasion, in that when the CD44v3–10 and CD44v8–10 isoforms were overexpressed individually in MCF10CA1h, only CD44v3–10, but not CD44v8–10, responded to OPN stimulation and promoted cancer cell invasion (Supplementary Figure 6B).

### CD44v3–10 rather than CD44s correlates with poor prognosis of breast cancer patients

**Figure 5:**
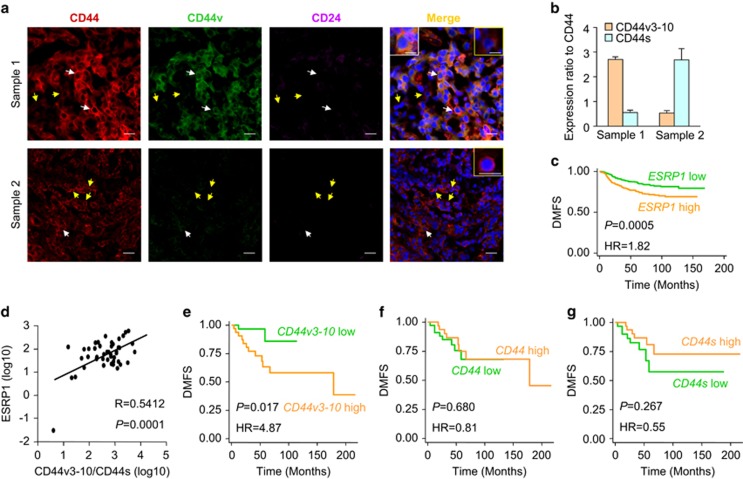
CD44v3–10 expression is positively correlated with poor prognosis in breast tumors. (**a**) Representative immunofluorescence analyses of total CD44, CD44v and CD24 in human breast cancer samples. White and yellow arrows denote CD24^−^/CD44^+^/CD44v^+^and CD24^−^/CD44^+^/CD44v^−^ cells, respectively. Insets in white and yellow boxes show representative CD24^−^/CD44^+^/CD44v^+^ and CD24^−^/CD44^+^/CD44v^−^ cells, respectively. (**b**) *CD44v3–10* and *CD44s* expression ratios analyzed by qPCR in human breast cancer samples, results are expressed as mean±S.D., *n*=3. (**c**) Distant metastasis-free survival (DMFS) analysis of the patients in the KM-Plotter database stratified by *ESRP1* expression, (*n*=1610). (**d**) Correlation of *ESRP1* expression and CD44v3–10/CD44s expression ratios in Qilu clinical samples (*n*=45). (**e–g**) Distant metastasis-free survival (DMFS) analysis of Qilu clinical samples stratified by expression levels of *CD44v3–10* (**e**), total *CD44* (**f**) and *CD44s* (**g**) (*n*=63). Scale bars, 10 *μ*m (insets) and 20 *μ*m (others)

Finally, we analyzed the clinical relevance of our findings. We first accessed the expression of CD24,CD44v and total CD44 in breast cancer clinical samples through immunofluorescence staining and observed both CD44v^+^ CSCs (CD24^−^/CD44^+^/CD44v^+^) and CD44v^−^ CSCs (CD24^-^/CD44^+^/CD44v^−^) in tumor tissues. Specifically, some samples contained predominantly CD44v^+^ CSCs, while CSCs in others were mainly CD44v^−^ (Figure 5a). In addition, the CD44v^+^ and CD44v^−^ CSCs preferred to express CD44v3–10 or CD44s, respectively (Figure 5b). These data confirmed the heterogeneity of CSCs in clinical samples. Furthermore, an analysis of the KM-Plotter breast cancer clinical database^50^ revealed that higher *ESRP1* expression was linked to poor prognosis of distant metastasis (Figure 5c). We further analyzed the correlation of different CD44 isoforms with metastasis in a cohort of breast samples collected from Qilu Hospital, and found that *ESRP1* expression was positively correlated to CD44v3–10/CD44s expression ratio (Figure 5d). More importantly, the expression of CD44v3–10, rather than CD44s and total CD44, was a prognostic factor of distant metastasis in these Qilu patients, as well as the KM-Plotter cohort (Figures 5e and g and Supplementary Figure 7A). The CD44v8–10 isoform, which was incapable for OPN binding due to the lack of V3–V7 exon area, was not prognostic of metastasis either (Supplementary Figure 7B), corroborating the conclusion that CD44v mediates cancer cell invasion in an OPN-dependent manner.

## Discussion

It is well conceived that CSCs are responsible for tumor initiation and recurrence at primary sites. However, the relationship between CSCs and tumor formation in secondary organs is less clear. Although studies have shown generally enhanced invasiveness and metastasis abilities of CSCs as compared to non-CSC populations, it may not necessarily be true that CSCs are uniformly more metastatic than non-CSCs. Instead, the observations could be explained by the existence of a subpopulation of highly metastatic cells in CSCs. Metastatic variants of CSCs was initially observed in pancreatic cancer and a CD133^+^/CXCR4^+^ subset of CSCs were shown to be essential for liver metastasis.^30^ Subsequently, CD26^+^ metastatic CSCs were reported in colorectal cancer.^31^ An additional study also showed that CD110^+^ and CDCP1^+^ CSCs of colorectal cancer led to liver and lung metastasis, respectively.^32^ However, metastatic CSCs were not identified in other types of cancers, posing the question whether the existence of metastatic CSCs is cancer type-specific. In this study we identify the metastatic subset of CSCs in breast cancer. As breast cancer is the most common cancer type in women and metastasis accounts for most of the cancer-related deaths, our study will have important clinical implication for cancer treatment. The theory of metastatic CSCs underscores the fact that primary tumor growth and spreading are distinct processes. Therefore, treatment of primary tumors and metastases requires therapeutic targeting of different molecules and different cancer cell populations.

The existence of metastatic and non-metastatic CSCs also highlights the heterogeneity of CSCs. Cancer cell heterogeneity makes any therapeutic approach targeting tumor bulks inefficient to kill all cancer cells and eventually treatment resistance is inevitable. The identification of CSCs has led to the optimistic proposal that targeting the real tumor-initiating populations of cancer cells will stop tumor recurrence. However, now we know CSC is also heterogeneous and thus CSC clearance will become a difficult task. It is conceivable that CSCs may be heterogeneous not only in metastatic capacities, but also in drug responses. Therefore, it is important to thoroughly study the heterogeneity of CSCs in order to effectively target these cells in therapeutics.

Notably, tumor metastasis is organ-specific and colonization of cancer cells in various distant organs has different prerequisites as the microenvironment differs. Gao *et al.* showed that distinct subpopulations of metastatic CSCs were responsible for colorectal cancer metastasis to liver and lungs.^32^ Here we only revealed the subpopulation of breast CSCs with enhanced capacity for lung metastasis. However, it is not known whether CD44v^+^ CSCs are also responsible for breast cancer metastasis to other organs. Therefore, further studies are needed to identify other organ-specific metastatic CSCs of breast cancer.

CD44 is widely used as a surface marker, especially together with CD24, to isolate CSCs from various solid tumors. However, the relationship between CD24^-^/CD44^+^ CSCs and distant metastasis has been ambiguous. Previous studies suggested that the prevalence of CD24^-^/CD44^+^ CSCs in breast tumors was linked to distant metastasis.^51^ In contrast, not all breast cancer cell lines containing high percentages of CD24^-^/CD44^+^ CSCs could give rise to lung metastasis.^27^ In addition, analyses of breast cancer samples showed that total CD44 expression can't predict distant metastasis efficaciously (Figure 5f and Supplementary Figure 7A). These seemingly contradictory results may be due to multiple isoforms of the CD44 protein. In this study we show that the variant isoforms, especially CD44v3–10, denote breast CSCs responsible for lung metastasis and correlate with clinical outcome. Therefore, it is necessary to distinguish CD44 isoforms in CSC studies, as well as in rational designing of clinical approaches for metastasis prognosis and CSC targeting.

Overall, we show that breast CSCs are heterogeneous and identify a subset of CSCs, characterized by *CD44v* and *ESRP1* expression, exhibiting the capacity of lung metastasis. Mechanistically, CD44v interacts with OPN in the lung microenvironment and promotes cancer cell invasion. These findings will enrich our understanding of CSCs in breast cancer and provide a rationale to target CSCs for treatment of breast cancer metastasis.

## Materials and Methods

### Plasmids and reagents

For shRNA knockdown of ESRP1, *CD44v6, CD44v7* and *CD44s*, the sense and antisense oligonucleotides were annealed and cloned into the BglII and HindIII site of pSUPER-retro-puro (OligoEngine, Seattle, WA, USA).^52^ For *ESRP1* overexpression, the human *ESRP1* cDNA was cloned into the pLVX-IRES-hygro vector with XbaI and BamHI digestion. All constructs were confirmed by sequencing. The sequences of primers and shRNA constructs were available in Supplementary Table S1. APC-anti-human CD24 (Biolegend311118, San Diego, CA, USA), FITC-anti-human CD44 (BD Pharmingen 555478, San Jose, CA, USA), mouse anti-human CD24 (Invitrogen MA5-11828, Carlsbad, CA, USA), rat anti-human CD44 (Santa Cruz sc-18849, Santa Cruz, CA, USA), rabbit anti-human CD44v7 (Millipore AB2083, Darmstadt, Germany), PE-goat anti-rabbit (Abcam ab97070, Cambridge, UK), FITC-goat anti-mouse (Proteintech SA0003-1, Chicago, IL, USA), CY3-donkey anti-rabbit (Biolegend 406402), AlexFluor647-goat anti-rat (Biolegend, 405416) antibodies and DAPI (Roche10236276001, Upper Bavaria, Germany) were used in this study for FACS and immunofluorescence analyses. Mouse anti-human CD44 (Cell Signaling Technology 3570, Danvers, MA, USA), rabbit anti-human GAPDH (Sigma G9545, St. Louis, MO, USA), rabbit anti-human ESRP1 (Santa Cruz sc-133945), rabbit anti-mouse Opn (Ruiying Biotechnology RLT3467, Suzhou, China) antibodies were used for Western blot assays. Recombinant human OPN (R&D systems 1433-OP-050, Minneapolis, MN, USA) was used to treat cancer cells in invasion assays. The Bouin's solution (Sigma HT10132) was used to fix lungs excised from mice.

### FACS analyses

Cells were analyzed on a Gallios analyzer (Beckman, Indianapolis, IN, USA) or sorted on a MoFlo Astrios Flow Cytometer (Beckman). Nonviable cells were excluded from further analyses. One million cells were incubated with 5 *μ*LAPC-anti-human CD24 (Biolegend 311118) and 20 *μ*l FITC-anti-human CD44 (BD Pharmingen 555478, San Jose, CA, USA) for 30 min at 4 °C. For CD24-CD44-CD44v6 triple antibody analysis, one million cells were incubated with 5 *μ*l APC-anti-human CD24 (Biolegend 311118), 20 *μ*l PE-anti-human CD44 (BD Pharmingen 555479) and 5 *μ*l FITC-anti-human CD44v6 (R&D FAB3660F, Minneapolis, MN, USA) for 30 min at 4 °C. To detect *CD44v7* expression, 500 thousands cells were incubated with 2 *μ*l rabbit anti-human CD44v7 (Millipore AB2083) for 30 min at 4 °C, followed by incubating with 1 *μ*l PE-goat anti-rabbit (Abcam ab97070) for 30 min at 4 °C. The data were analyzed with FlowJov10 (Tree Star, Ashland OR, USA).

### Tumorsphere culture

Cells were cultured as tumorspheres in DMEM/F12containing 20 ng/ml recombinant human EGF (R&D Systems, Minneapolis, MN, USA),10 ng/ml recombinant human bFGF (R&D Systems), 5 *μ*g/ml heparin sulfate (Sigma, H3149), 5 *μ*g/mL recombinant human insulin (Roche, Upper Bavaria, Germany), B27 supplement (Invitrogen 12587010) and 1% penicillin G-streptomycin (Invitrogen 15140-122). A total of 5000 cells were seeded in each well of a 6-well ultra-low attachment plate (Corning 3471, Corning, NY, USA). After two weeks of culture, spheres with diameters larger than 50 *μ*m were counted.

### Quantitative and semi-quantitative RT-PCR analyses

1 *μ*g of mRNA were s reverse-transcribed with a Primescript reverse transcriptase (Takara, Shiga, Japan). Semi-quantitative PCR was performed with the use of TaKaRa LA Taq (Takara), and PCR products were fractionated by agarose gel electrophoresis and stained with Goldview DNA dye. Quantitative PCR analysis was performed with a VII7A Real-time PCR System (Applied Biosystems, Waltham, MA, USA). The sequences of primers, including those for CD44 exon-specific qPCR, were available in Supplementary Table S1. For CD44 exon-specific qPCR, mRNA was subjected to genomic DNA depletion with DNase I (NEB, Ipswich, MA, USA) prior to reverse transcription.

### Trans-well invasion assays

A total of 5 × 10^4^ serum-starved cancer cells were resuspended in serum-free medium with or without 5 *μ*g/ml recombinant human OPN and seeded in the inserts (BD, 353504, San Jose, CA, USA) of 8 *μ*m pores with 3 mg/ml matrigel (BD, 354234). The inserts were placed in wells that contained media with 10% FBS for 24 or 48 h after seeding. Then the media were aspirated, and 200 *μ*l of trypsin was added into the wells to trypsinize the cells that had passed through the pores, followed by serum neutralization. The trypsinized cells were centrifuged for 30 min at 3000 r.p.m, resuspended in 30 *μ*l phosphate-buffered saline (PBS), and counted using a hemacytometer.

### Apoptosis assays

Cells were stained with Annexin V-APC/ 7-AAD apoptosis detection kit (KeyGEN, Nanjing, China) for 15 min in the dark at room temperature. Apoptosis was evaluated by flow cytometry Gallios (Beckman, Indianapolis, IN, USA) and apoptotic cells were defined as those that were positive for Annexin V and PI staining.

### Animal studies

All animal experiments were performed according to the guidelines for the care and use of laboratory animals, and were approved by the institutional biomedical research ethics committee of Shanghai Institutes for Biological Sciences. Female NOD/SCID or Balb/c athymic mice at the age of 6–8 weeks were used in all studies. Mice were grouped to ensure each group with equal body weight. The sample size was estimated according to prior experience of *in vivo* studies in the laboratory. Orthotopic injection and intravenous injection were performed to study primary tumor growth and lung metastasis as previously described.^52^ To study lung metastasis of mice orthotopically inoculated with MCF10CA1h or MCF10CA1a, primary tumors were surgically removed when reached the same size (1.5 cm^3^) and mice were sacrificed for lung metastasis evaluation by tumor nodule counting and H/E staining by a blinded observer. Since MCF10CA1a was successfully pre-labeled with luciferase, MCF10CA1a, lung metastasis was also analyzed by *ex vivo* bioluminescence imaging (BLI) with a NightOWL II LB 983 Imaging System (Berthold, Germany).

### Clinical analyses

Frozen breast tumor specimens were obtained from Qilu Hospital of Shandong University with informed patient consent and approval from the Institutional Review Board. Frozen tissues were used for RNA extraction, followed by qPCR analyses of *CD44v3–10, CD44v8–10, CD44s,* total *CD44*and ESRP1 expression levels. For distant metastasis-free survival analysis, the patients were classified into two groups according to the median expression level of each gene and patient survival was compared between the groups by Kaplan–Meier curves.

For immunofluorescence analysis, breast tumor tissues were embedded in O.C.T compound (Sakura Finetek, Tokyo, Japan) and sectioned into 6-*μ*m slides. Sections were blocked with PBS containing 0.2% Triton-X100 and 5% goat serum, and incubated with a primary antibody of CD24, CD44 or CD44v7 overnight at 4 °C. The specimens were washed with PBS for three times and incubated with a fluorochrome-conjugated secondary antibody. After washing, the samples were mounted with coverslips, followed by immunofluorescence analysis with the confocal microscopy Cell Observer (ZEISS, Oberkochen, Germany) and the ZEN blue edition software (ZEISS).

### Statistical analyses

Unless stated otherwise, results are presented as average±standard deviation in the figures. Two-tailed Student's *t*-test without assumption of equal variance was performed to compare the *in vitro* data. BLI curves were compared by ANOVA analysis. Nonparametric rank test was performed to compare the mouse lung metastasis nodules.

## Figures and Tables

**Table 1:** Tumor-initiating capacity of MCF10CA1h subpopulations

**No. of injected cells**	**10 000**	**1000**	**200**	**20**	**CSC frequency**	**P-values**	
CD24^-^/CD44^med^(P1)	10/10	10/10	14/24	0/14	1/238	P1 vs P2	0. 114
CD24^-^/CD44^hi^ (P2)	10/10	9/10	19/20	2/14	1/139	P1 vs P3	1. 38 × 10^-18^
CD24^+^/CD44^med^ (P3)	7/10	1/10	0/22	0/10	1/9101	P2 vs P3	4. 07 × 10^-26^
