# Supplementary material for: A CD44v+ subpopulation of breast cancer stem-like cells with enhanced lung metastasis capacity
Source: Cell Death Dis. 2017 Mar 16;8(3):e2679–. doi: 10.1038/cddis.2017.72 (PMC5386565; doi:10.1038/cddis.2017.72)
Supplement: Supplementary Information [file cddis201772x1.docx]

This supplementary file includes 7 supplementary figures and 1 supplementary table. Individual supplementary figure legend appears under the corresponding supplementary figure.

**Supplementary Figures**


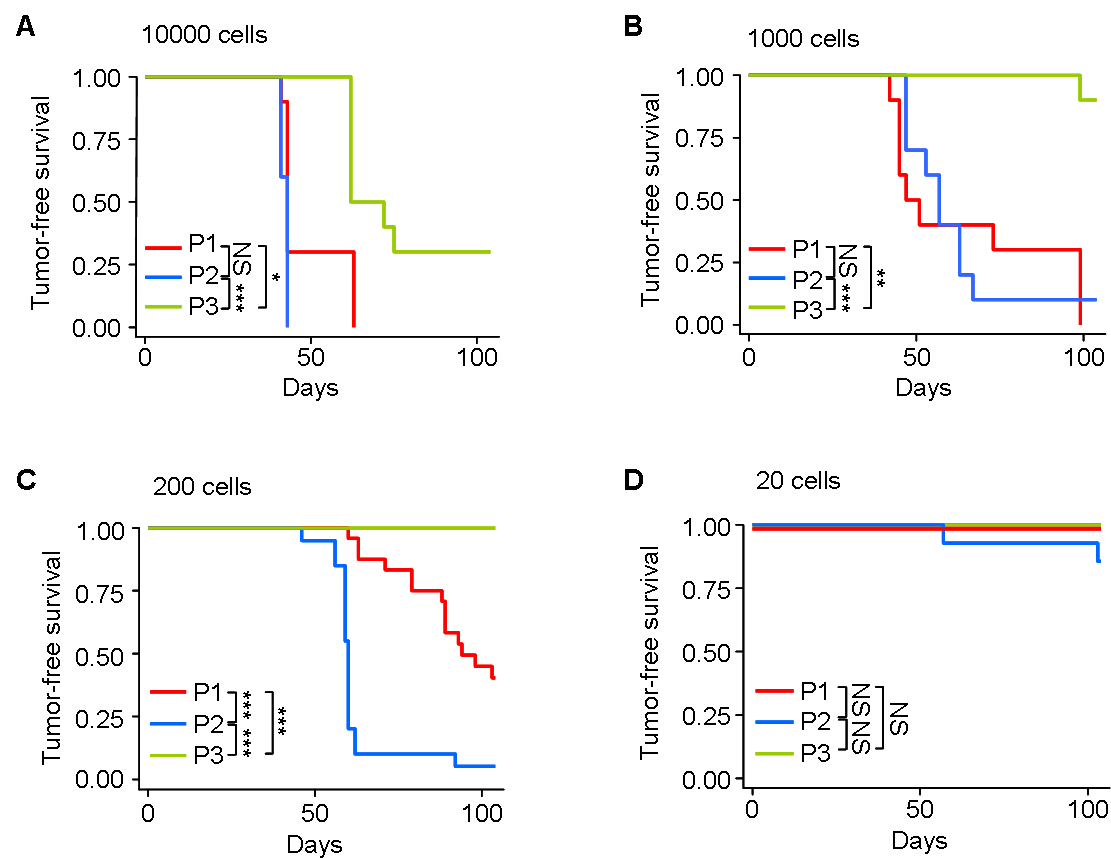


**Supplementary Figure 1. Tumor-free survival after orthotopic injection of MCF10CA1h CSC subpopulations. (A)** Tumor-free survival of mice injected with 10,000 cells (n=10 in each group). **(B)** Tumor-free survival of mice injected with 1,000 cells (n=10 in each group). **(C)** Tumor-free survival of mice injected with 200 cells (n≥20 in each group). **(D)** Tumor-free survival of mice injected with 20 cells (n≥10 in each group). * P<0.05, ** P<0.01, *** P<0.001. NS, not significant.

**Supplementary Figure 2. CD44v^+^ CSC subpopulations in breast cancer cell lines SCP28 (A), MDA-MB-231 (B) and BT20 (C).** Antibodies against CD24, CD44 and CD44v6 were used in flow cytometry. The cells were first analyzed for CD24 and CD44 expression (upper panels), and the CD24^-^CD44^+^ fraction was further analyzed for the CD44v status (lower panels). In upper panels, cells incubated without any antibody were used as negative control (NC); in lower panels, cells incubated with the CD44 antibody only (**A**, **B**) or no antibodies (**C**) were used as negative control. n = 3.


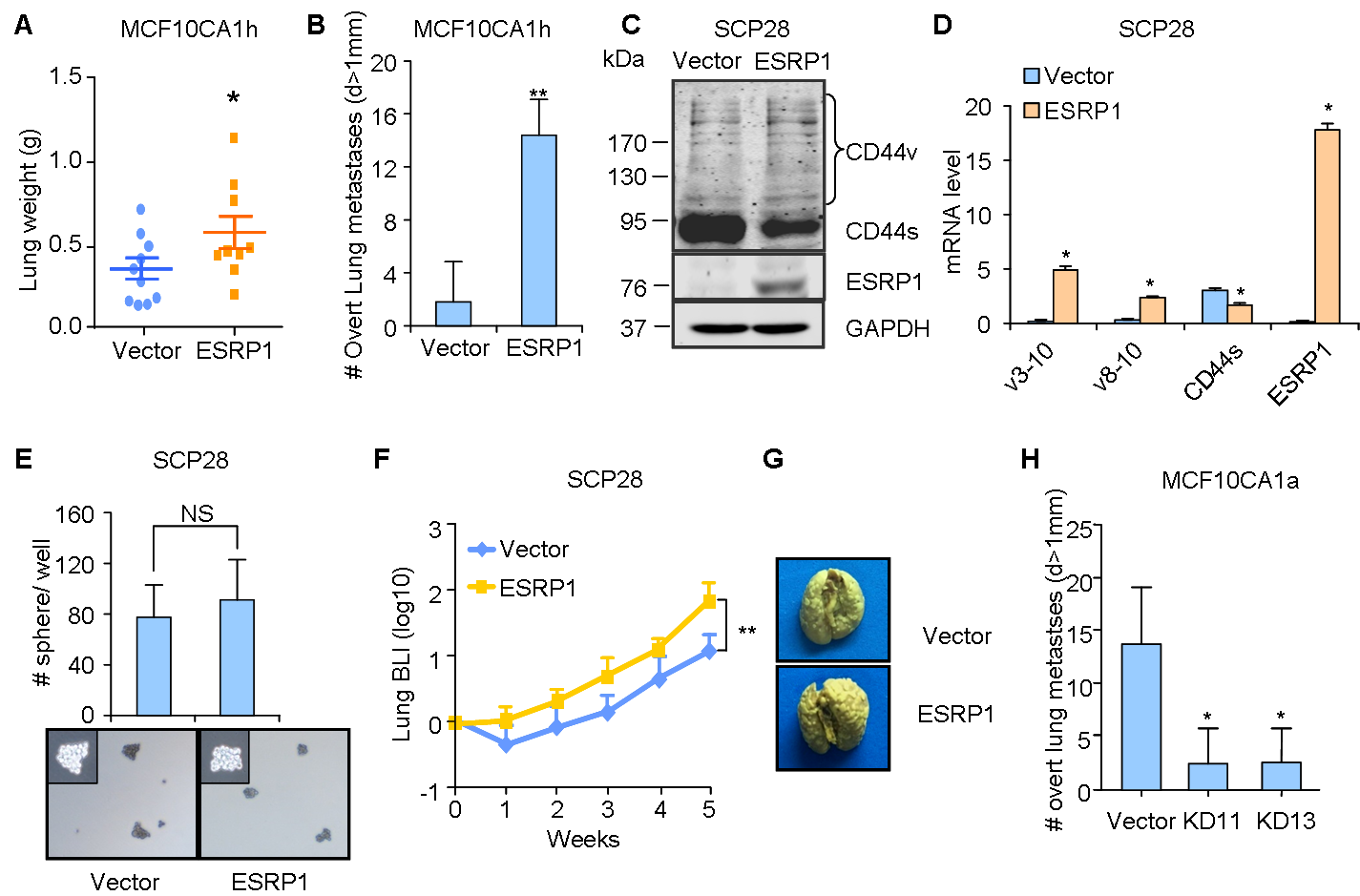


**Supplementary Figure 3. ESRP1 promotes breast cancer metastasis to lung without affecting cancer cell stemness. (A-B)** Lung metastasis after intravenous injection of MCF10CA1h with *ESRP1* overexpression (n≥10 in each group). Quantitation of lung weight, results are expressed as mean ± S.E.M **(A)** and overt lung metastases, results are expressed as mean ± SD **(B)** was shown. **(C-D)** Protein and mRNA expression of *CD44* isoforms and *ESRP1* in SCP28 after *ESRP1* overexpression, results are expressed as mean ± SD, n=3. **(E)** Quantization and representative images of tumor spheres in SCP28 after *ESRP1* overexpression, results are expressed as mean ± SD, n=3. **(F-G)** Lung metastasis after intravenous injection of SCP28 with *ESRP1* overexpression (n≥10 in each group). Quantitation of lung metastasis BLI, results are expressed as mean ± SD **(F)** and representative images of lungs **(G)** were shown. **(H)** Quantitation of overt lung metastases after intravenous injection of MCF10CA1a with *ESRP1* knockdown (n≥6 in each group), results are expressed as mean ± SD. * P<0.05, ** P<0.01, *** P<0.001. NS, not significant.


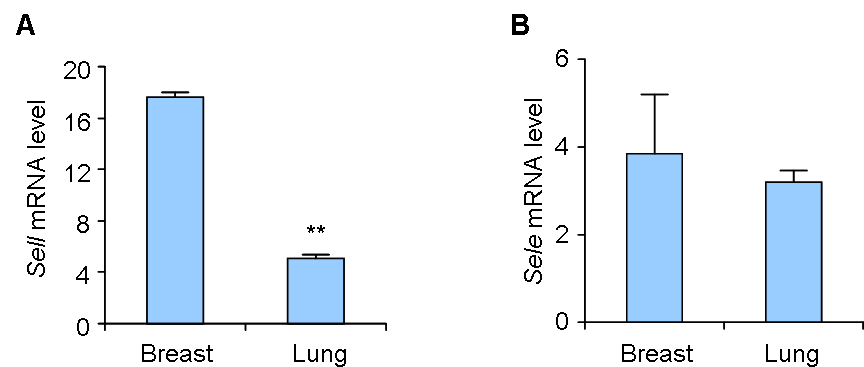


**Supplementary Figure 4. Expression of *Sell* and *Sele* in mouse breast and lung tissues.** **(A)** Expression of *Sell*, results are expressed as mean ± SD, n=3. **(B)** Expression of *Sele*, results are expressed as mean ± SD, n=3. * P<0.05, ** P<0.01.


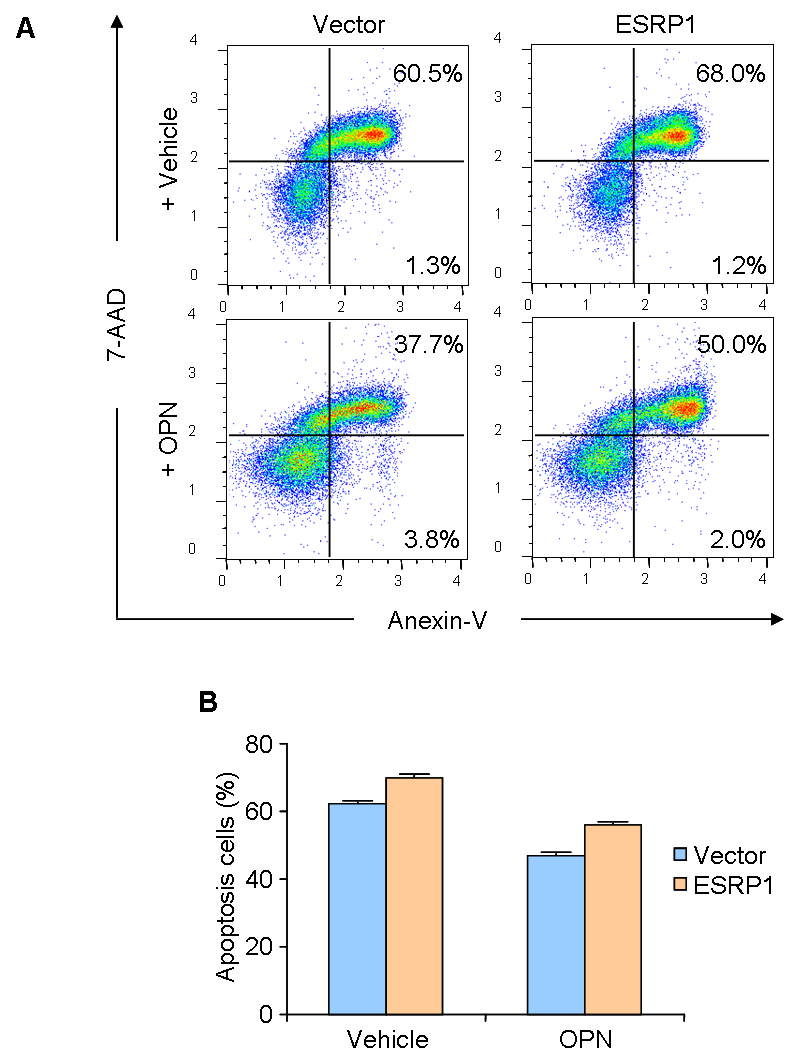


**Supplementary Figure 5. ESRP1 is not involved in OPN-mediated anti-apoptosis. (A)** Apoptosis of MCF10CA1h with *ESRP1* overexpression with or without the treatment of OPN (5ug/ml). **(B)** Statistics of the data in **(A)**, results are expressed as mean ± SD, n=3.


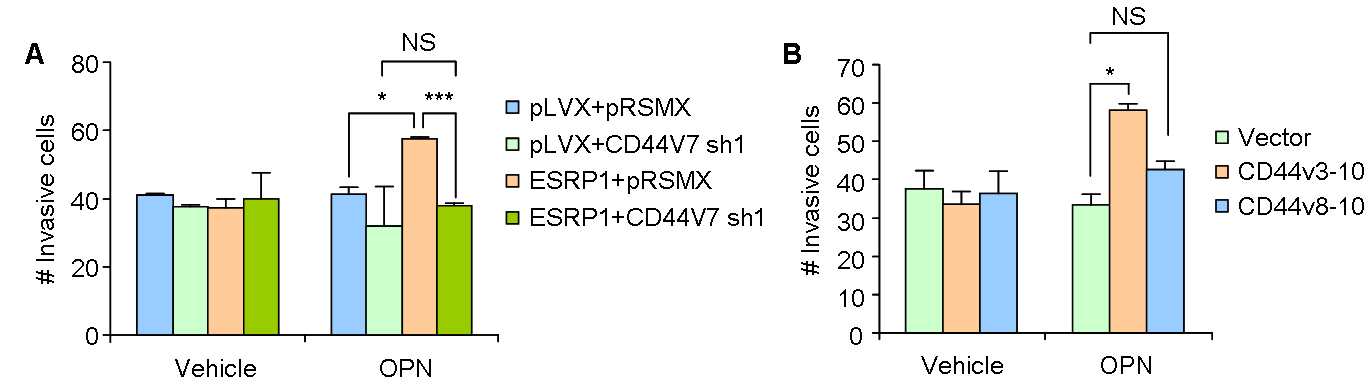


**Supplementary Figure 6. ESRP1 and OPN promote cancer cells invasion through CD44v3-v7. (A)** MCF10CA1h invasion with *CD44v7* knockdown and/or *ESRP1* overexpression, with or without the treatment of OPN (5 μg/ml), n=4. **(B)** MCF10CA1h invasion after *CD44v3-10* or *CD44v8-10* overexpression, with or without the treatment of OPN (5 μg/ml), n=4. Results are expressed as mean ± SD. * P<0.05, *** P<0.001, NS, not significant.


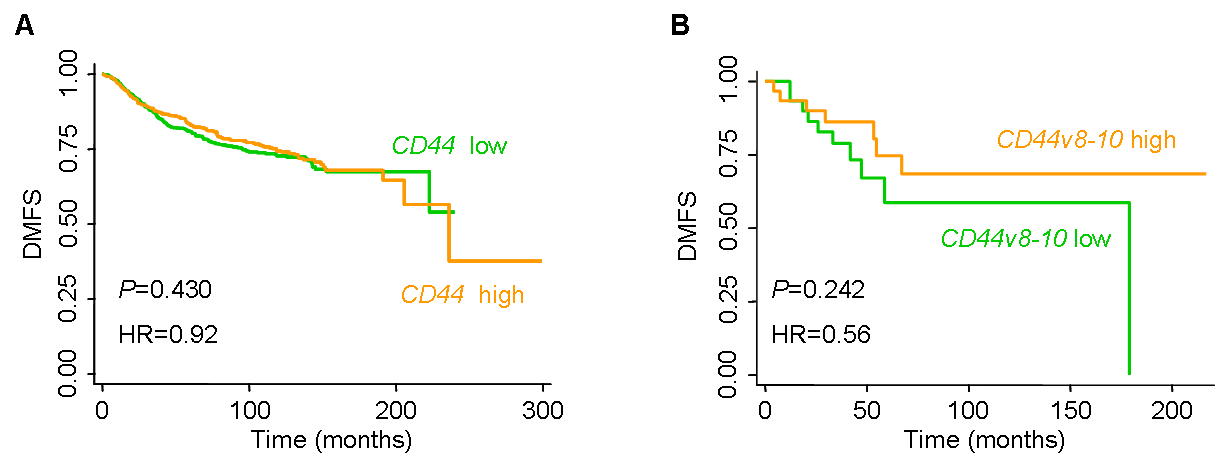


**Supplementary Figure 7. Total CD44 and CD44v8-10 are not effective prognostic factors of distant metastasis in breast tumors. (A)** Distant metastasis-free survival analysis of the patients in the KM-Plotter database stratified by total *CD44* expression (n=1610). **(B)** Distant metastasis-free survival analysis of Qilu clinical samples stratified by *CD44v8-10* expression (n=63).

| **Table S1: The sequences of primers and shRNAs used in study** | |
| --- | --- |
| Name | Sequences |
| ESRP1-KD11-sense | GATCTCCATAGGCAGTAATGCTTACTTTCAAGAGAAGTAAGCATTACTGCCTATTTTTTGGAAA |
| ESRP1-KD11-antisense | AGCTTTTCCAAAAAATAGGCAGTAATGCTTACTTCTCTTGAAAGTAAGCATTACTGCCTATGGA |
| ESRP1-KD13-sense | GATCTCCAGTAATGCTTACTACAATATTCAAGAGATATTGTAGTAAGCATTACTTTTTTGGAAA |
| ESRP1-KD13-antisense | AGCTTTTCCAAAAAAGTAATGCTTACTACAATATCTCTTGAATATTGTAGTAAGCATTACTGGA |
| CD44v6-KD-sense | GATCTCCGGCAACTCCTAGTAGTACATTCAAGAGATGTACTACTAGGAGTTGCCTTTTTGGAAA |
| CD44v6-KD-antisense | AGCTTTTCCAAAAAGGCAACTCCTAGTAGTACATCTCTTGAATGTACTACTAGGAGTTGCCGGA |
| CD44v7-KD-sense | GATCTCCAGAGGACAGTTCCTGGACTTTCAAGAGAAGTCCAGGAACTGTCCTCTTTTTTGGAAA |
| CD44v7-KD-antisense | AGCTTTTCCAAAAAAGAGGACAGTTCCTGGACTTCTCTTGAAAGTCCAGGAACTGTCCTCTGGA |
| CD44s-KD-sense | GATCTCCCCAGAGACCAAGACACATTTCAAGAGAATGTGTCTTGGTCTCTGGTTTTTGGAAA |
| CD44s-KD-antisense | AGCTTTTCCAAAAACCAGAGACCAAGACACATTCTCTTGAAATGTGTCTTGGTCTCTGGGGA |
| ESRP1-PCR-F | GCTCTAGAATGACGGCCTCTCCGGATTACT |
| ESRP1-PCR-R | CGGGATCCGGGCCCTTAAATACAAACCCATTC |
| CD44-RTPCR-F | GTGATGGCACCCGCTATGTCCAG |
| CD44-RTPCR-R | CACTGGGGTGGAATGTGTCTTGGTC |
| GAPDH-RTPCR-F | AAGGCTGGGGCTCATTTGCAG |
| GAPDH-RTPCR-R | CCAAATTCGTTGTCATACCAGG |
| CD44c5-qPCR-F | GCAACCCTACTGATGATGACG |
| CD44c5-qPCR-R | TCTGGGATGGGGTGTACAGT |
| CD44v2-qPCR-F | TGCTACAGCAACTGAGACAGG |
| CD44v2-qPCR-R | TGTGTGAAGATGATTCTTTGACTC |
| CD44v3-qPCR-F | GGGAGCCAAATGAAGAAAATG |
| CD44v3-qPCR-R | TGGTGCTGGAGATAAAATCTTC |
| CD44v4-qPCR-F | ACACCACGGGCTTTTGAC |
| CD44v4-qPCR-R | CATCCTTGTGGTTGTCTGAAGTA |
| CD44v5-qPCR-F | ACTGCTTATGAAGGAAACTGG |
| CD44v5-qPCR-R | GTGCTTGTAGAATGTGGGGT |
| CD44v6-qPCR-F | CAACGGAAGAAACAGCTACC |
| CD44v6-qPCR-R | CTGTTGTCGAATGGGAGTCT |
| CD44v7-qPCR-F | CAGCCTCAGCTCATACCAGC |
| CD44v7-qPCR-R | GCTTGATGACCTCGTCCCAT |
| CD44v8-qPCR-F | GGACTCCAGTCATAGTATAACGC |
| CD44v8-qPCR-R | CATTGAAAGAGGTCCTGTCCT |
| CD44v9-qPCR-F | AGCAGAGTAATTCTCAGAGCTT |
| CD44v9-qPCR-R | TGCTTGATGTCAGAGTAGAAGT |
| CD44v10-qPCR-F | TGTCACAGGTGGAAGAAGAGA |
| CD44v10-qPCR-R | GAGGTCACTGGGATGAAGGT |
| CD44c15c16-qPCR-F | CCCATACCACTCATGGATCTG |
| CD44c15c16-qPCR-R | GGTGTCCTTATAGGACCAGAGGT |
| CD44v3-10-qPCR-F | ACAGACAGAATCCCTGCTACCAGTA |
| CD44v3-10-qPCR-R | CTCTTTCATCTTCATTTTCTTCATTTG |
| CD44v8-10-qPCR-F | AGACAGTCCCTGGATCACCGA |
| CD44v8-10-qPCR-R | GTTATACTATGACTGGAGTCCATATTGG |
| CD44s-qPCR-F | ACAGACAGAATCCCTGCTACCAGAGA |
| CD44s-qPCR-R | GCCACTGTTGATCACTAGCTTTTTC |
| CD44-qPCR-F | GACACCATGGACAAGTTTTGG |
| CD44-qPCR-R | CGGCAGGTTATATTCAAATCG |
| ESRP1-qPCR-F | CAATATTGCCAAGGGAGGTG |
| ESRP1-qPCR-R | GTCCCCATGTGATGTTTGTG |
| GAPDH-qPCR-F | GAAGGTGAAGGTCGGAGTC |
| GAPDH-qPCR-R | GAAGATGGTGATGGGATTTC |
| Opn-qPCR-F | GAAAGGGCAGCCATGAGTC |
| Opn-qPCR-R | TGGAATGCTCAAGTCTGTGTG |
| Sell-qPCR-F | TGGTCATCTCCAGAGCCAAT |
| Sell-qPCR-R | GCAGTCCATGGTACCCAACT |
| Sele-qPCR-F | CAAATCCCAGTCTGCAAAGC |
| Sele-qPCR-R | ACATTTCATGTTGCCCTGCT |
| Gapdh-qPCR-F | TCCCACTCTTCCACCTTCGATGC |
| Gapdh-qPCR-R | GGGTCTGGGATGGAAGTGGTGAGG |
